# An Enquiry into the Nature and Causes of Epilepsy; with the Function of the Spleen and Thyroid Body

**Published:** 1842-07-01

**Authors:** 


### AN ENQUIRY INTO THE NATURE AND CAUSES OF EPILEPSY; WITH THE FUNCTION OF THE SPLEEN AND THYROID BODY. By *John Jackson*, M.R.C.S. 8vo. pp. 107. Whittaker and Co. 1842.

THE above is certainly a curious title. The conjunction of the three subjects will sound oddly in antiquated ears, who have rarely associated epilepsy with spleen and thyroid gland—we beg pardon, body. We agree with our author that, because the uses of the two organs in question have not been yet discovered, it does not follow that they are inscrutable; but we certainly think that it does (though he says it does *not)* "justify the supposition that they are necessarily *less obvious* than those of other organs." Surely the office of the spleen is infinitely less apparent than that of its neighbour—the stomach, or even of the liver? But let that pass. We shall not go over a description of the epileptic paroxysm with Mr. Jackson, as very few are unacquainted with the phenomena; but we shall quote the following passage for the comfort of homicides, suicides, parricides, &c. who may contemplate an easy death by the rope or the handkerchief.

"The symptoms of an epileptic paroxysm bear a close resemblance to those observed in asphyxia from suspension. When a criminal is executed, or an animal is hung, the following phenomena are produced:—unconsciousness—convulsions, chiefly of the face and upper extremities—turgescence and lividity of countenance—relaxation of sphincters—seminal emission—and death. Precisely the same group of symptoms, therefore, as occur in a fatal epileptic paroxysm. But suppose that a person attempts suicide by suspension, and is only *half* hung, —that he is cut down before life is extinct, and recovers—the analogy still holds; he has no recollection of having suffered during the suspension. The following quotation is from Dr. Elliotson's Lectures : 'Although the individual may struggle, and although he may be all but dead, and may hang so long as to be insensible, it does not appear that there is any suffering.' " 13.

We have some doubts on this point. The first execution we ever witnessed was that of a deserter from a ship of war, who plunged overboard and attempted to join the rebels encamped on one side of Waterford harbour. He was captured and taken to Newfoundland, where he was tried and hanged. In his struggles, he broke the cords by which his arms were bound, and instantly grasped the rope round his neck, and endeavoured to prevent the strangulation ! In this horrible and agonizing state he hung for nearly half an hour, when his struggles ceased ! We shall not easily forget such a scene, which had nothing in it to make us enamoured of the halter or handkerchief, however lauded these may be by physiologists as first rate modes of euthanasia.

Our author, observing the analogy between asphyxia and the epileptic paroxysm, was led to suspect that the pathological state of both was the same—namely, simple cerebral congestion. To ascertain the truth or error of this theory, he placed a ligature on the superior cava of a dog, above the vena azygos. Great lividity and turgescence of the tongue and mucous membrane of the mouth, with complete insensibility, immediately ensued ; but no convulsions. The cords being loosed, the dog got up—shook himself—and walked to the other end of the room. He was killed by a drachm of hydrocyanic acid, and, on examination, the ligature was found perfectly tight. This experiment appeared to our author to prove that cerebral congestion, *per se,* produces insensibility, but not convulsions. The thought then occurred that the latter might be owing to spinal congestion. Three fourths of the blood from the vertebral sinuses being returned to the heart by the cava superior, Mr. Jackson determined to tie the vena azygos as well as the superior cava. A dog was again selected, and a ligature passed round both vessels. Insensibility the most complete, accompanied by strong tetanic convulsions of the whole muscular system immediately ensued. There was also great livor and turgescence of the mucous membrane of the tongue, lips, and mouth. All these symptoms continued till the animal's death, which quickly followed. Another similar experiment was made, with the same result. From these experiments our author thinks he may draw the following conclusions.

"1. That cerebrebal congestion gives rise to insensibility, and spinal congestion to convulsions.

2. As the presence of cerebro-spinal congestion in the epileptic paroxysm is denoted by the lividity and turgescence of the face and hands; and as insensibility and convulsions are the two essential symptoms of the paroxysm,—that cerebro-spinal congestion is the pathological state or proximate cause which gives rise to those symptoms.

3. That the primary cause, the cause *par excellence,* or primum mobile of the epileptic seizure, is necessarily a *something* which is capable of rapidly inducing cerebro-spinal congestion." 18.

Cerebro-spinal congestion, then, is regarded as the *proximate cause* of epilepsy. But the question arises, what is it that gives rise to this cerebro-spinal congestion ? Or what is the nature of this primary cause ?

"There are, I conceive, at least two ways in which the ingress of blood into the auricle from the superior cava may be suddenly prevented, and cerebro-spinal congestion consequently induced. Firstly, blood being returned to the heart of a *quality* more or less incapable of stimulating its right cavities, and which the ventricle therefore refuses to propel or very imperfectly propels, through the lungs; the immediate effect of which necessarily is, that distension more or less excessive of the right cavities is produced, and the further ingress of blood from both venæ cavæ either greatly impeded, or altogether denied: and secondly, the *quantity* of blood supplied to, and the force with which it enters the auricle by the *inferior* auricular opening, being suddenly and temporarily augmented by a force greater than that with which the blood descends into that cavity from the superior cava; and which sudden and temporary augmentation can only be effected by an inordinate rush of blood from the hepatic veins: then, in this case as in the former, the right cavities, but more especially the auricle, would suffer undue distension, ingress by the superior cava would be prevented, and cerebro-spinal congestion would ensue, and endure so long as the rush into the auricle from the hepatic veins continued." 21.

Our author enters into a long disquisition on the physiology of the portal circulation, which seems to present great difficulty, being a kind of anomaly, where the *vis a tergo* from the heart and arteries propels the blood through two venous and two capillary systems. Our author's arguments and reasonings are ingenious and plausible; but they are too long to quote entire, and cannot be condensed without mutilation. The following passage brings us to the goal at which Mr. J. has arrived.

"This leads to the further inference, that a regulating as well as propelling power is required:—for the due performance of so important a function there should be an organ; and there is; an organ to which the real function has not hitherto been ascribed, and that organ is the Spleen. It is the spleen which propels the portal blood through the portal trunk, branches, plexuses, and hepatic veins, to the inferior cava and auricle, and regulates the quantity and force with which it is propelled." 27.

Mr. J. attaches a good deal of importance to "the resemblance of the spleen, in texture and phenomena, to the erectile tissues," and especially to the placenta, both in structure, in function, and in disease.

"Having remarked the similarity in the external appearance and internal organization of the spleen and the placenta; that the latter and the umbilical vein perform previously to birth the function of the former and the portal vein; and that the spleen, and therefore the placenta, in texture and phenomena bear a remarkable resemblance to the erectile organs, the most important of which is the penis; and having moreover observed that the spleen and the placenta, in addition to their elasticity, possess a peculiar contractile power, by which power both are enabled to propel the blood through the capillaries of the liver and the hepatic veins to the right auricle; we are now prepared to expect that this *contractility* alike possessed by each of these organs, the penis, spleen, and placenta, will be excited by the same agencies, whether of a moral or physical nature." 36.

This is all very ingenious, and, we have no doubt, quite convincing—to the mind of the writer; but whether it will be so to that of the reader, is another question. As the whole essay is anatomico-physiological—with no trifling dash of the conjectural, we must refer our readers to the work itself for as pretty a piece of special pleading as we have for a long time seen. The essay gives us a very high opinion of Mr. Jackson's acquirements in those two paramount branches of our profession—anatomy and physiology. And when years shall have furnished him with ample experience and observation, we predict that he will, one day, contribute some valuable information in the practical department of medicine.

